# Oxidation-Reduction Potential in Patients undergoing Transcatheter or Surgical Aortic Valve Replacement

**DOI:** 10.1155/2018/8469383

**Published:** 2018-11-14

**Authors:** Kathrin Heldmaier, Christian Stoppe, Andreas Goetzenich, Ann-Christina Foldenauer, Rachat Zayat, Thomas Breuer, Gereon Schälte

**Affiliations:** ^1^Department of Anesthesiology, Medical Faculty, RWTH Aachen University, Aachen, Germany; ^2^Department of Intensive and Intermediate Care, Medical Faculty, RWTH Aachen University, Aachen, Germany; ^3^Department of Cardiothoracic Surgery, Medical Faculty, RWTH Aachen University, Aachen, Germany; ^4^Fraunhofer Institute for Molecular Biology and Applied Ecology (IME), Branch for Translational Medicine and Pharmacology (TMP), Frankfurt am Main, Germany

## Abstract

**Background:**

Aortic valve stenosis has gained increasingly more importance due to its high prevalence in elderly people. More than two decades ago, transcatheter aortic valve replacement emerged for patients who were denied surgery, and its noninferiority has been demonstrated in numerous studies. Oxidative stress has generated great interest because of its sensitivity to cell damage and the possibility of offering early hints of clinical outcomes. The aim of the present study was to investigate whether there is a significant difference between transcatheter (TAVR) or surgical aortic valve replacement (SAVR) in terms of the changes in oxidation-reduction potential (ORP) and antioxidant capacity. Therefore, we investigated perioperative oxidative stress levels and their influence on clinical outcomes.

**Methods:**

A total of 72 patients (50% TAVR versus 50% SAVR) were included in the present study. Static oxidation-reduction potential (sORP) and antioxidant capacity were measured using the RedoxSys™ Diagnostic System (Luoxis Diagnostics, USA) in serum samples drawn before and after surgery, as well as on the first postoperative day. In addition, clinical data were obtained to evaluate the clinical outcome of each case.

**Results:**

TAVR patients had higher preoperative sORP levels compared to the SAVR patients and more severe comorbidities. Unlike the TAVR cohort, patients in the SAVR group showed a significant difference in sORP from the pre- to postoperative levels. Capacity demonstrated higher preoperative levels in the SAVR cohort and also a greater difference postoperatively compared to the TAVR cohort. Regression analysis revealed a significant correlation between pre- and postoperative capacity levels (r = -0.9931, p < 0.0001), providing a method of predicting postoperative capacity levels by knowing the preoperative levels. According to the multivariable analysis, both sORP and antioxidant capacity are dependent on time point, baseline value, and type of surgery, with the largest variations observed for time effect and surgery method.

**Conclusion:**

A high preoperative sORP level correlated to more severe illness in the TAVR patients. As the TAVR patients did not show significant differences in their preoperative levels, we assume that there was a smaller production of oxidative agents during TAVR due to the less invasive nature of the procedure. Baseline values and development of antioxidant capacity values strengthen this hypothesis. The significant correlation of pre- and postoperative capacity levels might allow high risk patients to be detected more easily and might provide more adequate and individualized therapy preoperatively. This trial is registered with clinicaltrials.gov, identifier: NCT 02488876.

## 1. Introduction

In our aging society, physicians must treat diseases of the elderly. The incidence of aortic valve stenosis is increasing rapidly among the elderly, and it is associated with a high mortality rate. In 2006, the prevalence among patients older than 75 years was 4.6% [1].

Surgical aortic valve replacement (SAVR) is still the standard procedure for patients with severe aortic stenosis [2, 3]. Thus, in a prospective study, Iung et al. found that 33% of patients included in the Euro Heart Survey were not scheduled for SAVR, primarily due to age and low left ventricular ejection fraction [4].

In recent decades, transcatheter aortic valve replacement (TAVR) has emerged as a promising alternative to conventional surgery [5–7]. In a recent meta-analysis, the mortality rate and symptomatic improvement were found to be similar or even better with the transcatheter approach compared to conventional procedures [7]. This less invasive intervention results in a shorter intervention time, reduced mechanical ventilation time, an overall decrease in hospitalization, and shorter stays in intensive care units [8, 9]; therefore, it is an adequate option for high risk patients.

Today, TAVR is the standard procedure for patients with high [2, 3, 10] or prohibitive/highly increased surgical risk [2, 3, 6], and there are efforts to widen its field of application to patients with intermediate surgical risk [5].

Little is known about the oxidative stress response during minimal invasive transcatheter operations, particularly compared to the classical surgical procedures. Oxidative stress, per se, is known to modulate cellular pathways and gene expression. Cells either respond by adapting to oxidative stress or through cell death or uncontrolled proliferation. This process has been shown to lead to diseases [11] and organ dysfunction [12].

Its measurement has gained increasingly more attention in recent decades as an additional parameter to explain the reason for the incidence of postoperative nonsurgical complications. For example, associations have been observed between high oxidative stress and acute [13–15] and chronic kidney disease [16, 17], as well as acute lung injury [18].

Assuming that the height of oxidative stress levels might serve as a predictor of both severity of diseases and clinical outcomes, many studies have been performed. Bar-Or and Rael found a significant association between oxidation-reduction potential and outcomes after acute severe brain injury [19], as well as for oxidative stress and sepsis [20].

In the present study, we focused on the measurement of static oxidation-reduction potential (sORP) and antioxidant capacity in patients undergoing either surgical or transcatheter aortic valve replacement. Our aim was to investigate whether a correlation between the overall oxidation-reduction potential and clinical outcome could be identified.

## 2. Methods

### 2.1. Patients

In a prospective observational single center trial, 80 patients who underwent either SAVR or TAVR were included between November 2011 and May 2016. All patients voluntarily participated in the study and provided written consent; the study protocol conformed to the ethical guidelines of the 1975 Declaration of Helsinki, and the study was approved by the local ethics committee (Rheinisch-Westfälische Technische Hochschule (RWTH) Aachen University, Faculty of Medicine, Ethical board, EK 151/09).

The inclusion criterion was an elective intervention, according to German federal medical regulations. The exclusion criteria were endocarditis, pericarditis, combined surgery (e.g., replacement of ascending aorta, coronary artery bypass graft), and all malignant diseases.

**Figure 1:**
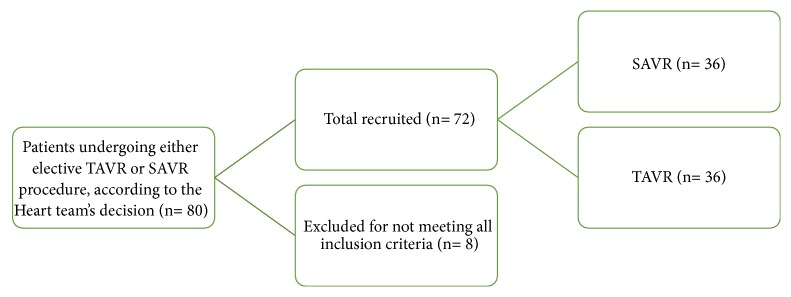
A STROBE flowchart demonstrating patient recruitment.

The decision whether to perform SAVR or TAVR was made by the local interdisciplinary heart team (including cardiac surgeons, cardiologists, and cardiac anesthesiologists), respecting physician experience and the European System for Cardiac Operative Risk Evaluation II (EuroScore Study Group 2011), according to guidelines [2, 3]. The participant recruitment process is shown in Figure 1. As the results of the EuroScore II increase with older age, comorbidities, and female sex, there was a set preselection of patients, in terms of the type of surgery that they received.

### 2.2. Blood and Data Acquisition

Venous blood samples were collected directly before (T1) and after surgery (T2), as well as on the first postoperative day, 24 h after surgery (T3). After preparing and centrifuging them at 2,500 rpm for 10 minutes, the serum was collected and immediately frozen for storage.

In addition to the 30-day survival rate and ICU and hospital length of stay (LOS), postoperative complications (e.g., death, bleeding, administration of postoperative noradrenalin, and need for blood transfusions) were documented. Chronic kidney disease (CKD) was defined as a progressive loss of renal function for at least three months, according to the KDIGO guidelines [21], and acute kidney injury (AKI) was a urine output of less than 0.5 ml/kg body weight/h for at least 6 hours, a creatinine increase of 0.3 ml/dl for at least 48 hours, or a creatinine increase 1.5 times the baseline value [22].

### 2.3. Measurement

Measurement of the static oxidation-reduction potential (ORP) was performed using the RedoxSYS™ Diagnostic System (Luoxis Diagnostics, Inc., Englewood, CO, USA), which measures the overall integrated redox potential of a biological sample, in this case serum. The RedoxSYS™ Diagnostic System was used following the manufacturer's instructions by applying 20 *µ*l of serum on each testing strip. The results are provided as static ORP (sORP) [mV] and antioxidant capacity [*µ*C]. The sORP offers an overview of all, known and unknown, oxidant and reductant components. Higher values indicate the presence of more oxidative stress. Antioxidant capacity is a marker of the antioxidant reserve, representing the ability of an organism to respond to oxidative stress [23].

Clinical data were collected, and further parameters were assessed in the local laboratory.

### 2.4. Statistical Analysis

In our study, we compared the influence of SAVR and TAVR procedures on the development of the stress parameter sORP and the antioxidant capacity. All continuous variables are expressed as the mean ± standard deviation (SD). For heavily skewed distributions the median, the 0.25-quantile (Q1), and the 0.75-quantile (Q3) were used instead. Categorical variables are expressed as absolute frequencies and percentages. Patient characteristics were compared between the surgical methods (TAVR versus SAVR), with the help of two-sided unpaired t-tests. If the data were skewed, an exact Wilcoxon rank-sum test was used instead. Comparisons between frequencies were conducted using the Fisher exact test. For these tests, exact odds ratios (OR) and corresponding 95% (penalized) likelihood confidence intervals (CI) were reported. Correlations between the continuous influence parameters and primary endpoints were assessed with Pearson's correlation coefficient. As the capacity change from baseline was not normally distributed and strongly dependent upon the size of the baseline measurement, regression analysis was performed to evaluate the impact of the baseline measurement, and Wilcoxon tests were chosen to compare the capacity difference at different time points.

A linear model with repeated measures was applied to investigate the change in sORP over time. Therefore, the difference in the sORP measurement from later time points (end of surgery/24h after surgery) to the preoperative sORP baseline measurement (“sORPdiff”) was modeled as the outcome. The baseline sORP measurement, gender, time (end of surgery/24h after surgery), surgery method (TAVR versus SAVR), surgery method (by time interaction), and baseline (by surgery method interaction) were considered to be fixed effects in a multivariable model. A random intercept was modeled for each patient. Several other models were fitted; however, the selected one was the best, according to the AIC value (Akaike information criterion). Age and BMI were neglected, as both effects are correlated with the surgery method but showed no significant impact in a univariate sensitivity analysis. Furthermore, the exclusion of these effects improved the model, according to the AIC value. A Kenward-Rogers adjustment was used to account for the small sample size, and an unstructured covariance matrix was assumed. For the post hoc tests, a Tukey adjustment was performed.

Except for minor differences, the same statistical model as that for the sORP change was applied to analyze the capacity over time. Instead of the difference from baseline, the actual capacity measurement from later time points (end of surgery/24 h after surgery) were modeled as outcomes and logarithmized to meet the model requirements. Similar to the sORP analysis, the baseline capacity measurement was considered to be a fixed covariate. Except for the exclusion of the surgical method by the time interaction effect, the remaining factors and covariance structure were chosen in concordance with the sORP model. Note that the capacity change from baseline could not be modeled as an outcome, as the residuals of the fitted models were not normally distributed independent of the chosen covariance structure, considered effects, and possible data transformation.

For both repeated measure analyses, regression estimates with SD and p values are reported.

We assessed any effect in the statistical models as significant if the corresponding p value fell below the 5% margin. As this was an explorative study, no alpha adjustment was performed. Boxplots were chosen to present the data distribution of sORP over time. Statistical analysis was performed using SAS for Windows, Version 9.4 (SAS Institute, Cary, NC, USA); “Proc Mixed” was used for the repeated measure analysis.

## 3. Results

### 3.1. Baseline Characteristics

In total, 72 patients were included, in accordance with the inclusion criteria. The participants ranged in age from 53 to 93 years (mean, 77.3 ± 9.2 years). There were 40 female and 32 male patients. The baseline characteristics of all patients are shown in Table 1. One patient with a recommendation for TAVR due to a high EuroScore II of 51% refused the TAVR procedure, despite the clear recommendation of the heart team, and had a successful SAVR. As we had assumed, the TAVR collective was significantly older compared to the SAVR group (83 ± 5.3 years versus 71.5 ± 8.8 years; p < 0.001), and it included significantly more women than men (69.4% versus 41.7%, p = 0.0321, OR = 3.18) and showed a significantly higher percentage of chronic renal insufficiency (30.6% versus 5.6%, p = 0.012, OR = 7.48). Moreover, they showed a higher level of dyspnea, as measured by the NYHA score. While patients in the SAVR group widely suffered from dyspnea NYHA I or II (58.3% SAVR versus 25.0% TAVR), the score was ≥ III in the TAVR patient group (41.7% SAVR versus 75.0% TAVR). The TAVR patients showed a higher risk for NYHA III or IV compared to the SAVR patients (p = 0.008; OR 4.2 with 95%-CI= [1.38; 13.07]).

### 3.2. Surgery, Intensive Care Unit Stay, Survival

The surgery duration was significantly longer in the SAVR group compared to the TAVR group (182 ± 50.9 minutes versus 85.5 ± 34.9 minutes, p< 0.001). No significant difference was found in relation to the length of stay (LOS) in the ICU; however, the SAVR patients had a longer overall hospital LOS compared to the TAVR patients (16.1 ± 11.5 days versus 10.4 ± 4.3 days, p= 0.011).

The 30-day survival rate was equal in both groups (mortality rate, 5.56%). From each group, one patient died because of septic shock. The second patient in the TAVR group died on the fifth postoperative day, after a high risk valve-in-valve procedure (EuroScore II 30.52%); in the SAVR group, an additional patient died, most likely due to gastrointestinal bleeding, although no autopsy was performed to confirm this cause.

### 3.3. sORP and Antioxidant Capacity

**Figure 2:**
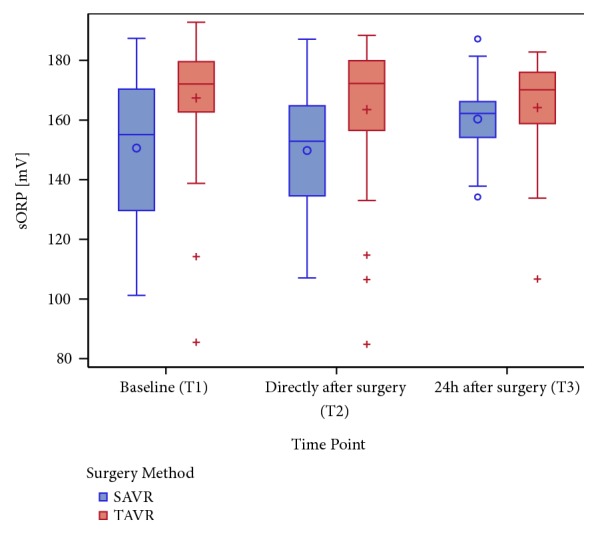
Boxplot of sORP in mV at all time points (categorical presentation is on the x-axis: T1, T2, and T3), categorized by surgery methods (SAVR versus TAVR). Note that the baseline sORP in the TAVR cohort is significantly higher compared to that of the SAVR group.

**Figure 3:**
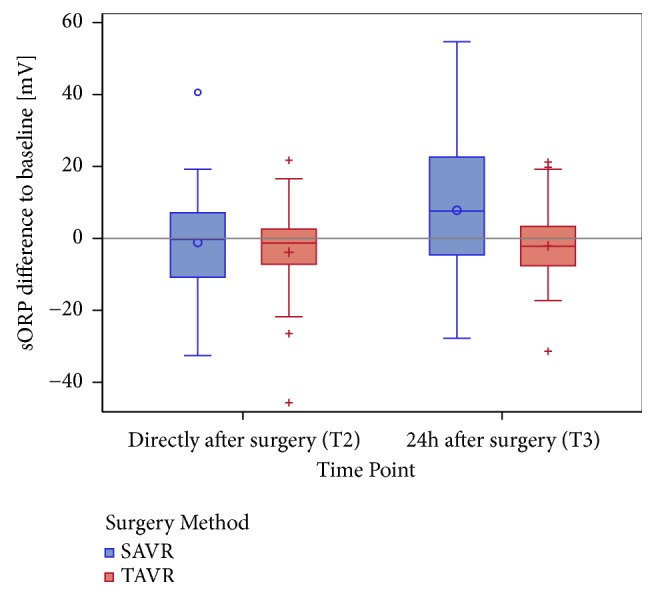
Boxplot of sORP differences at T2 compared with T3 and baseline T1 (i.e., T2-T1, T3-T1), in mV (the categorical presentation is on the x-axis), stratified by the surgery method (SAVR versus TAVR). The reference line at 0 indicates the increase or decrease in contrast to baseline (T1).

The baseline measurement of redox status revealed significantly higher sORP (p = 0.0034) and lower antioxidant capacity levels in the TAVR patients (p = 0.0235) (Table 1, Figure 2). Regarding the time course of the sORP levels over time for the whole study population, no significant changes were observed. Interestingly, dividing patients according to their type of intervention, the sORP levels of the TAVR patients remained roughly stable, whereas there were significant differences in the baseline value of the patients in the SAVR group (Figure 3).

**Figure 4:**
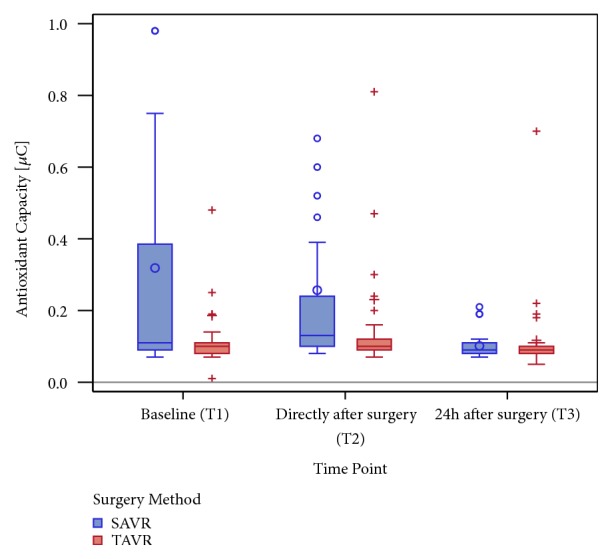
A boxplot of the antioxidant capacity in *µ*C at all investigated time points (the categorical presentation is on the x-axis: T1, T2, and T3) stratified by surgery method (SAVR versus TAVR) and truncated at 1.0 *µ*C for better illustration of the data distribution. Only 6 capacity measurements were observed above 1.0 *µ*C: 2 measurements in the SAVR group with values < 2.0 *µ*C each at T1 and T2; 2 measurements in TAVR: 2.72 *µ*C at T1 and 3.37 *µ*C at T2.

Antioxidant capacity shows higher preoperative levels in the SAVR cohort (SAVR 0.11 *µ*C (Q1: 0.09; Q3: 0.39) versus TAVR 0.10 *µ*C (Q1:0.08; Q3: 0.11); p = 0.0235), fitting the observation that preoperative sORP levels were lower in this group compared to the TAVR group. Consistent with the change of sORP during the observed time, a greater difference of postoperative values of antioxidant capacity compared to TAVR (Figure 4) was observed.

To investigate a correlation between the preoperative capacity level and capacity difference compared to baseline for both postoperative measurements (T2 and T3), regression analysis was performed. The results revealed an association between the observed parameters, and the association was particularly strong between baseline and capacity difference at 24 hours after surgery (T3) (r = -0.9931, p < 0.0001). The regression Capacity_diff (24 h postsurgery) = 0.06509 – 0.79664*∗*Baseline_Capacity offered a possibility of predicting postoperative capacity levels 24 hours after surgery. The coefficient of determination was R^2^ = 0.9863.

### 3.4. Association of Redox Potential with Clinical Data

**Figure 5:**
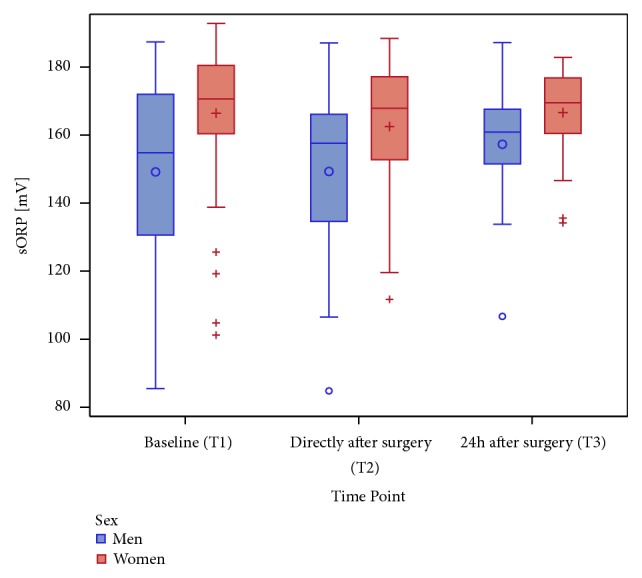
A boxplot of sORP in mV at all time points (categorical presentation on x-axis: T1, T2, and T3) stratified by sex (male versus female).

First, a gender-specific difference was observed. Women showed generally higher sORP levels than men, which is consistent to the observation that the TAVR group includes more women than men (T1: p (adjusted) = 0.084; T2: p (adjusted) = 0.037; T3: p (adjusted) = 0.037, according to the Wilcoxon rank-sum test) (Figure 5).

**Figure 6:**
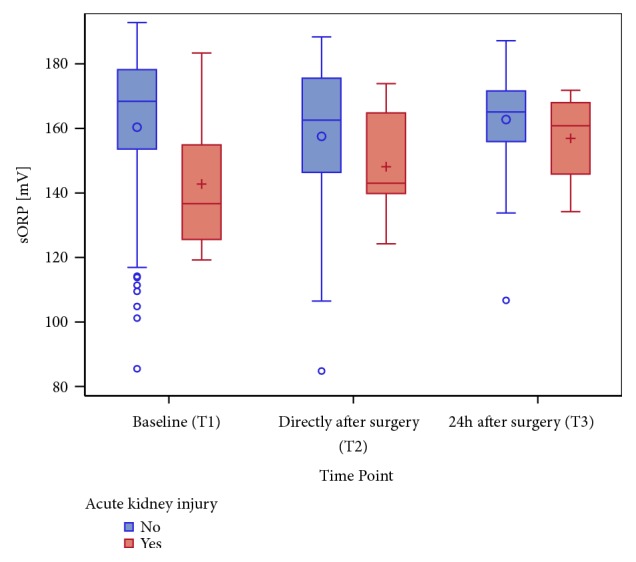
A boxplot of sORP in mV at all time points (the categorical presentation is on the x-axis: T1, T2, and T3), stratified by the status of acute kidney injury (AKI) at the corresponding time point.

sORP values were lower at each observed time point for the patients who developed a postoperative AKI. However, particularly at T2, increasing sORP values showed an association with the postoperative development of AKI, as defined by the KDIGO [22]. The increase of sORP was associated with the new onset AKI, as shown in Figure 6. There was no significant association between postoperative AKI and the type of intervention, although a weak trend might be observed (SAVR 13.9% versus TAVR 2.8%, p = 0.1987).

The need for (packed) red blood cell transfusions/units (PRBC) and postoperative treatment with noradrenalin was significantly higher in the patients treated by SAVR (PRBC: SAVR 55.56% versus TAVR 25%, p= 0.0156; noradrenalin: SAVR 94.4% versus TAVR 22.2%, p < 0.0001). Nevertheless, the need for PRBC units was not significantly correlated to sORP (T1-T3: p adj. = 0.487). In contrast, patients receiving postoperative noradrenalin showed lower sORP levels than those without therapy, at each measurement (T1 – T3: p adj. = 0.0183).

The type of intervention did not show a significant correlation to postoperative bleeding (SAVR 8.3% versus TAVR 25.0%, p = 0.1113, OR = 3.67), and there was no significant difference in the sORP levels (T1: p adj. = 0.7778; T2: p adj. = 0.3105; T3: p adj. =0.7778).

In multivariate analysis the sORP change from baseline depended significantly on the baseline value, time points, and type of surgery (Table 2). In particular, the baseline by surgical method interaction effect was significant (p = 0.0011), revealing an increased sORP change over time, if the baseline sORP measurement was increased; the increase depended upon the applied surgical method. Gender did not affect the sORP change. Looking at the regression slopes, the largest variation was explained by the surgical method (-44.5579 ± 14.3210) and the time effect at 24 h after surgery (9.7183 ± 2.8079) (Table 2). Thus, after adjusting the fixed parameters in a linear model, the SAVR procedure was still associated with a higher change in sORP.

Similar to sORP, antioxidant capacity depended on the baseline value, time points, and type of intervention, according to the multivariable analysis (Table 3). In particular, the baseline by surgery method interaction effect was significant (p = 0.0027), revealing an increased capacity change over time if the baseline capacity measurement was increased, with the increase depending on the applied surgery method. Gender did not affect the capacity change (Table 3).

Looking at the regression slopes, the largest variation was explained by the surgery method (0.6163 ± 0.1672) and time effect (-0.4339 ± 0.06493), showing larger effects than the baseline capacity values. Hence, the complex linear model confirms that the SAVR patients demonstrated higher capacity differences than the TAVR patients and that postoperative capacity levels are highly associated with preoperative baseline levels.

## 4. Discussion

In this trial, we could demonstrate that patients who underwent the TAVR procedure presented higher pre- and postoperative levels of sORP. In contrast to the SAVR group, these values remained stable in the TAVR group, without any major changes, whereas significant differences from the pre- to postoperative sORP levels were measured in the SAVR group. For both interventions, the preoperative sORP levels were independently associated with postoperative differences in the sORP levels. In addition, the peri- and postoperative increase of sORP led to the development of postoperative AKI. In addition, the administration of postoperative noradrenalin was associated with lower sORP levels at all time points. There was no correlation between sORP and death; the need for PRBC units or postoperative bleeding was found.

Antioxidant capacity, which decreased in the presence of reactive oxygen species, supported those findings. Preoperatively, patients in the SAVR group showed higher values of antioxidant capacity compared to patients in the TAVR group. While the pre- and postoperative levels of antioxidant capacity did not show significant differences in the TAVR group, a significant decrease was observed in the SAVR group. A regression analysis revealed not only a strong association between the pre- and postoperative values but also the nearly perfect ability of preoperative antioxidant capacity values to predict postoperative values. The multivariable model revealed that the sORP and antioxidant capacity changes depended on the baseline value, time points, and type of surgery, with the largest variation due to time effect and surgery method.

As previously reported, the sORP levels both pre- and postoperatively were higher in the TAVR group. Patients in the TAVR group were generally older than the SAVR patients and showed numerous and severe comorbidities resulting in a higher surgical risk, suggesting that they were suitable candidates for TAVR [2, 3]. For example, chronic renal insufficiency appeared significantly more often in this group compared to the SAVR group. There is evidence for chronic kidney disease contributing to higher oxidative stress levels and vice versa, oxidative stress enhances the progression of the disease, and, therefore, this result can be reasonably explained [16, 17]. Moreover, chronic kidney disease is frequently associated with cardiovascular diseases, with a 10- to 20-fold higher annual mortality rate compared to general population [24].

Older age contributes to elevated levels of oxidative stress [25, 26]. In 1996, Sohal and colleagues hypothesized that higher levels of oxidative stress in the elderly might be generated by increased numbers of reactive oxygen metabolites, low levels of antioxidant agents, and a lack of repair or removal mechanisms [27]. As reported, the TAVR cohort had a significantly higher mean age compared to the SAVR cohort. In this trial, we confirmed that higher sORP levels, as measured in the TAVR group, are related to multiple comorbidities, particularly chronic kidney disease and higher age. Conversely, patients in the SAVR cohort were mostly younger and had fewer comorbidities than those in the TAVR group. Thus, they presented with lower sORP levels, both pre- and postoperatively.

Interestingly, the postoperative levels of sORP showed greater differences to the preoperative values in the SAVR than in the TAVR group. The same effect was observed regarding capacity: while the SAVR patients showed higher antioxidant capacity levels, indicating a higher capability of resisting oxidative damage, this value changed more after surgery in this group than in the TAVR group.

We conclude that these findings indicate a higher production of reactive oxygen species during open heart surgery compared to the transcatheter approach. This finding might be due to less tissue damage and, therefore, less production of reactive species. Our findings support previously published data indicating the less invasive and damaging nature of TAVR compared to conventional surgery [8, 9].

This thesis is supported by shorter operation times, a decreased need for blood transfusions, and postoperative noradrenalin administration and a shorter overall hospitalization, compared to SAVR. Unlike the trials by Zierer and Conradi, no differences in the length of ICU stay were observed between the two cohorts. Nevertheless, in our study, the overall hospitalization was shorter in the TAVR group compared to the SAVR group. As Zierer et al. compared TAVR with minimally invasive partial sternotomy, this explanation might reasonably explain the contradicting data. Nevertheless, differences compared to the results of Conradi et al. cannot be fully explained and should be investigated more closely. In accordance with their data, the TAVR patients had shorter operation times [8, 9]. Conradi et al. reported shorter ventilation times for TAVR patients [8]. In contrast, the TAVR patients in our trial underwent surgery using local plus conscious sedation, without endotracheal intubation. Our findings may emphasize the Conradi results underlining the less traumatic approach, with a reduced need for mechanical ventilation.

The observed postoperative complications, death, bleeding, and, correspondingly, the need for PRBC units and acute kidney injury, must be investigated. Both death and bleeding had similar rates in each group, and no association between these complications and sORP/antioxidant capacity levels was observed. Blood transfusions occurred more frequently in the SAVR group; however, there was no association between the need of transfusions and sORP. This indicates a higher blood loss during surgery or more severe postoperative bleeding in the SAVR group. The need for postoperative noradrenalin application was higher among the SAVR patients; patients receiving noradrenalin showed lower sORP levels.

As previously mentioned, patients developing a postoperative AKI had lower sORP levels. However, a higher incidence of AKI was observed in patients with a notable increase of sORP after surgery. In our study, no significant differences between the development of postoperative AKI and the type of intervention were observed, although a trend towards a higher percentage of postoperative AKI was observed in the SAVR patients. According to Bellomo et al., various factors contribute to the development of acute kidney injury after cardiac surgery: exogenous and endogenous toxins, metabolic factors, ischemia and reperfusion, neurohormonal activation, inflammation, and oxidative stress. The use of cardiopulmonary bypass has been identified as a possible/facultative risk factor for SAVR, while nephrotoxic agents (e.g., contrast agents), used in the TAVR procedure, have a negative impact and should be avoided [15]. As both interventions are related to damaging factors, no significant difference of cardiac surgery associated AKI could be proven, although our trial indicates a higher exposure in SAVR patients. Nevertheless, it has to be mentioned that we could only identify six patients with AKI; thus, the observed results should be interpreted with caution.

The strong association of pre- and postoperative capacity levels and its ability to predict the postoperative redox state and, therefore, likely worse clinical outcomes seem to be highly promising. The association might provide physicians, even before surgery, with an indication of how a patient could cope with oxidative agents produced during surgery and, therefore, might provide better preoperative and individualized therapy. Further investigation is needed to explain this effect more effectively.

Recently, malondialdehyde, a biomarker of oxidative stress, was proven to be a promising predictor of adverse events for patients undergoing either surgical or transcatheter aortic valve replacement (in a ROC-analysis) [28]. In the present trial, investigating the overall redox potential instead of one biomarker, the occurrence of potential adverse events could not be predicted.

In cardiothoracic patients, Stoppe et al. investigated the levels of antioxidant trace elements, such as selenium, zinc, and copper, before surgery and after ICU admission. In alignment with our results, a postoperative decrease of antioxidant trace elements and the ability of low postoperative selenium levels to predict multiorgan failure were demonstrated [29]. Subsequently, a study investigating the effects of administration of sodium selenite perioperatively was performed; however, no elevation of selenium levels at the first postoperative day and no change in postoperative outcomes were shown [30].

A recent clinical trial, suggesting oxidative stress as a promising target for therapy, has been conducted, substituting antioxidants to reduce oxidative damage. Red raspberry and vitamins C and E are suggested to possess beneficial effects in reducing oxidative stress [31–33].

Interest in this investigation is high and appears promising, particularly keeping in mind the possible benefit of treating high risk patients preoperatively in an adequate manner after identifying them thoroughly. Further study is needed to prove the effectivity and clinical benefit of such an individualized therapy.

### 4.1. Study Limitations

The primary study limitations are the nonrandomization of patients and the monocentric trial design. The investigated parameters were consistent to previous investigation, although we were able to include a larger study population [28]. Nevertheless, this work should be regarded as a pilot study, showing interesting trends that should be investigated in a larger population size. In addition, the samples were frozen, and the measurements were conducted over a two-week period, accepting that the different storage times might lead to changes in the values. To minimize this effect, samples were centrifuged within 30 minutes after being drawn and then constantly frozen at -80°C. According to Luoxis, this process does not affect the reliability of samples [34]. Despite the lack of reliable data, we acknowledge that the used comedications may have significantly contributed to the extent of redox stress measured in the patients.

## 5. Conclusion

In the present study, we were able to show differences in the sORP and capacity levels of patients undergoing either transcatheter or conventional surgical intervention. The TAVR patients with higher and lower preoperative levels of sORP compared to the SAVR cohort continued to have high but stable levels of sORP (and vice versa, low antioxidant capacity levels) after the intervention. The SAVR patients, however, showed large changes in the sORP and capacity levels from their pre- to postoperative measurements. We conclude that these significant differences are a result of high intraoperative production of reactive oxygen species during conventional surgery. Both sORP and capacity appeared to depend greatly on the surgical method and time of measurement. A wider range of sORP and capacity difference to baseline has been observed in SAVR patients. The good correlation of pre- and postoperative capacity levels can be used to identify high risk patients and adapt existing preoperative treatment adequately to their special needs. Nevertheless, it should be considered that the measured redox parameters are not fully established at the current time. These data encourages future studies to validate the clinical significance of these results in an adequately powered cohort of cardiac surgical patients.

## Figures and Tables

**Table 1:** Baseline characteristics. BMI: body mass index; BSA: body surface area; sORP: static oxidation-reduction potential; EuroSCORE: European System for Cardiac Operative Risk Evaluation ^*∗*^p < 0.05.

	**All patients **	**SAVR**	**TAVR**	**Comparison SAVR vs. TAVR**
	**n = 72**	**n = 36**	**n = 36**	**p-value** **(t-test/Wilcoxon Test/Fisher Test)**
**Gender (male)**	32 (44.4%)	21 (58.3%)	11 (30.6%)	0.0321^*∗*^
**Age (years)**	77.3 ± 9.2	71.5 ± 8.8	83.0 ± 5.3	<0.0001^*∗*^
**Weight (kg)**	76.3 ± 18.0	82.1 ± 16.8	70.5 ± 17.5	0.0054^*∗*^
**BMI**	27.4 ± 5.7	28.4 ± 5.1	26.5 ± 6.2	0.0481^*∗*^
**BSA (m** ^**2**^ **)**	1.9 ± 0.2	2.0 ± 0.2	1.8 ± 0.2	0.0005^*∗*^
**Obesity (BMI > 30)**	21 (29.2%)	13 (36.1%)	8 (22.2%)	0.2997
**Hypertension (yes)**	58 (80.6%)	29 (80.6%)	29 (80.6%)	1.0000
**Smoker (yes)**	7 (9.7%)	5 (13.9%)	2 (5.6%)	0.0254^*∗*^
**Nonsmoker but former smoker (yes)**	14 (19.4%)	11 (30.6%)	3 (8.3%)	
**Diabetes mellitus (yes)**	28 (38.9%)	15 (41.7%)	13 (36.1%)	0.8092
**Chronic renal insufficiency (yes)**	13 (18.1%)	2 (5,6%)	11 (30.6%)	0.0120^*∗*^
**Logistic EuroScore**	16.1 ± 10.3	10.5 ± 9.6	21.7 ± 7.5	<0.0001^*∗*^
**sORP baseline [mV]**	166.3 (Q1: 142.8; Q3: 178.2)	155.2 (Q1:129.7; Q3: 170.4)	172.1 (Q1: 162; Q3: 179.6)	0.0034^*∗*^
**Antioxidant capacity baseline [**µ**C]**	0.1 (Q1: 0.1; Q3: 0.2)	0.11 (Q1: 0.09; Q3: 0.39)	0.10 (Q1:0.08; Q3: 0.11)	0.0235^*∗*^

**Table 2:** Target variable: the sORP difference to baseline from the end of surgery time point (reference category of time measurement). SAVR is used as a reference category for the surgery method. ^*∗*^p < 0.05.

**Linear mixed model of the sORP difference from baseline**					
	**Estimate**	**SD (Estimate)**	**Num DF/Den DF**	**F-value**	**p-value**
**Intercept**	75.9354	9.2095	70.7	8.25	<.0001^*∗*^
**Baseline sORP**	-0.5068	0.05984	66.9	-8.47	<.0001^*∗*^
**Surgery Method **(TAVR)	-44.5579	14.3210	63.6	-3.11	0.0028^*∗*^
**Gender **(female)	-1.3573	2.2735	62.1	-0.60	0.5527
**Time Point (overall)**			1/67.2	8.14	0.0059^*∗*^
24 h in the ICU	9.7183	2.8079	68.2	3.46	0.0009^*∗*^
**Time by Surgery Method Interaction** (24 h in the ICU*∗*TAVR)	-8.2360	3.9399	67.2	-2.09	0.0404^*∗*^
**Baseline (sORP) by Surgery Method Interaction** (Baseline*∗*TAVR)	0.3022	0.08787	60.2	3.44	0.0011^*∗*^

**Table 3:** Target variable: capacity difference from baseline from the end of surgery time point (reference category of time measurement). SAVR is used as the reference category for the surgery method. ^*∗*^p < 0.05.

**Linear mixed model for capacity difference to baseline**					
	**Estimate**	**SD (Estimate)**	**Num DF/Den DF**	**F-value**	**p-value**
**Intercept**	-1.1308	0.1145	86.4	-9.88	<.0001^*∗*^
**Baseline log. capacity**	0.3893	0.05237	61.8	7.43	<.0001^*∗*^
**Surgery Method **(TAVR)	0.6163	0.1672	55.7	3.69	0.0005^*∗*^
**Gender **(female)	-0.06455	0.06559	51.8	-0.98	0.3296
**Time Point (overall)**	-0.4339	0.06493	63.2	-6.68	<.0.0001^*∗*^
**Baseline (Capacity) by Surgery Method Interaction** (Baseline*∗*TAVR)	0.2444	0.07787	56.7	3.14	0.0027^*∗*^

## Data Availability

The data used to support the findings of this study are available from the corresponding author upon request.
